# Borna disease.

**DOI:** 10.3201/eid0302.970205

**Published:** 1997

**Authors:** C G Hatalski, A J Lewis, W I Lipkin

**Affiliations:** University of California, Irvine 92697-4290, USA.

## Abstract

Borna disease virus, a newly classified nonsegmented negative-strand RNA virus with international distribution, infects a broad range of warm-blooded animals from birds to primates. Infection causes movement and behavioral disturbances reminiscent of some neuropsychiatric syndromes. The virus has not been clearly linked to any human disease; however, an association between infection with the virus and selected neuropsychiatric disorders has been suggested. We reviewed recent advances in Borna disease virus research, focusing on evidence of infection in humans.


### Borna Disease

**Carolyn G. Hatalski, Ann J. Lewis, and W. Ian Lipkin**
University of California, Irvine, California, USA

Borna disease virus, a newly classified nonsegmented negative-strand RNA virus with international distribution, infects a broad range of warm-blooded animals from birds to primates. Infection causes movement and behavioral disturbances reminiscent of some neuropsychiatric syndromes. The virus has not been clearly linked to any human disease; however, an association between infection with the virus and selected neuropsychiatric disorders has been suggested. We reviewed recent advances in Borna disease virus research, focusing on evidence of infection in humans.

Although Borna disease was first recognized in the early 1800s as a neurologic syndrome with an infectious basis, Borna disease virus (BDV) has only recently been characterized as the causative agent. BDV is the prototype of a newly recognized virus family, *Bornaviridae*, within the nonsegmented negative-strand RNA viruses (order *Mononegavirales*) (1,2).

The molecular biology of the virus has several unusual aspects, including nuclear localization for replication and transcription (3), overlap of open reading frames and transcription units (4,5), posttranscriptional modification of subgenomic RNAs (6,7), and marked conservation of coding sequence across various animal species and tissue culture systems (8,9). BDV replicates at lower levels than most known viruses (10,11), is not lytic, and persists in the nervous system despite a vigorous immune response. In the classic syndrome, infected animals exhibit movement and behavior disorders (10,12,13); however, clinical signs may be dramatic, subtle, or inapparent depending on the integrity and intensity of the host immune response to viral gene products (14).

### Natural Infection and Transmission

Originally described as a disease of horses, Borna disease has also been found in sheep, llamas, ostriches, cats, and cattle (15). Because an even larger variety of species has been infected experimentally, the host range is likely to include all warm-blooded animals; no data exist concerning infection of species other than warm-blooded hosts.

Address for correspondence: W. Ian Lipkin, Laboratory for Neurovirology, Department of Neurology, University of California, Irvine, CA 92697-4290; fax: 714-824-1229; e-mail: ilipkin@uci.edu.

The geographic distribution of BDV is unknown. Natural infection has been reported only in Central Europe, North America, and parts of Asia (Japan and Israel). However, this apparent geographic restriction may reflect lack of reliable methods and reagents for diagnosis of infection or failure to consider the possibility of BDV infection. Recent reports of asymptomatic naturally infected animals suggest that the virus may be even more widespread than previously thought (16-18).

Neither the reservoir nor the mode of transmission of natural infection is known. An olfactory route for transmission has been proposed because intranasal infection is efficient, and the olfactory bulbs of naturally infected horses show inflammation and edema early in the course of disease (19,20). BDV nucleic acid and proteins in peripheral blood mononuclear cells (PBMC) also indicate a potential for hematogenous transmission (21-28). Experimental infection of rodents results in virus persistence and is associated with the presence of viral gene products in saliva, urine, and feces (28); such secreta/excreta are important in the transmission of other pathogenic viruses (e.g., lymphocytic choriomeningitis virus and hantaviruses). Thus the rodent provides the potential for both a natural reservoir and vector; however, because natural BDV infection has not been reported in rodents, their role in BDV transmission to other domesticated animals and humans remains speculative. No data concerning vertical transmission of BDV in natural or experimental hosts have been published.

### Animal Models for BDV Pathogenesis

Borna disease in naturally infected horses and sheep is characterized by agitated aggressive behavior that progresses over weeks to paralysis and inanition (29). Because its immune-mediated disease most closely resembles that in naturally infected horses and sheep, the Lewis rat has been selected as a rodent model. Rats infected as adults exhibit hyperactivity and exaggerated startle responses coincident with viral gene products in limbic system neurons and infiltration of mononuclear cells into the brain (13,30). The inflammation recedes over several weeks, but the virus persists and animals show stereotyped motor behavior, dyskinesias, and dystonias associated with distinct changes in the central nervous system (CNS) dopamine system (12,31), as well as decreased activity and cachexia (13). In contrast, rats infected as neonates have a disease characterized by stunted growth, hyperactivity, subtle learning disturbances, and altered taste preferences and do not mount a cellular immune response to the virus (32,33).

Behavioral disturbances have been reported in experimentally infected primates: tree shrews and rhesus monkeys. Infected tree shrews have altered social and sexual behavior, manifested as abnormal dominance relationships and failure to mate (34). Infected rhesus monkeys are initially hyperactive and subsequently become apathetic and hypokinetic (35).

### BDV and Neuropsychiatric Disease

### Serology (Table 1)

Its broad host and geographic range suggests that BDV might cause human neuropsychiatric disease. Because the behavioral disturbances in animals resembled those of affective disorders, particularly bipolar depression, initial studies investigated these disorders. The earliest work to suggest a link between BDV and human mental illness came from a serologic survey in 1985 of 285 patients with affective disorders in the United States, 694 psychiatric patients in Germany, and 200 healthy controls (36). An indirect immunofluorescence assay (IFA) was used to detect antibodies reactive with a BDV-infected cell line; sera from 12 (4.3%) patients from the United States and four (< 1%) patients from Germany were immunoreactive; no sera from controls were immunoreactive. Sera from many of these patients were subsequently analyzed by a Western immunoblot assay based on BDV nucleoprotein (N) and phosphoprotein (P) purified by affinity chromatography from infected rabbit kidney cells (37). In this study of 138 patients with affective disorders and 117 healthy controls, antibodies to N were found in 53 (38%) patients versus 19 (16%) controls; antibodies to P were found in 16 (12%) patients versus five (4%) controls; antibodies to both proteins were found in nine (6.5%) patients versus one (< 1%) control.

Table 1. Serum immunoreactivity to Borna disease virus in various diseases

| Disease | Prevalence: Disease | (%) | Controls | (%) | Assay | Ref. |
|---|---|---|---|---|---|---|
| Psychiatric (various) | (4/694) | 0.6 | (0/200) | 0 | IFA | (36) |
| | (13/642) | 2 | (11/540) | 2 | IFA | (60) |
| | (200-350/ 5000) | 4-7 | (10/ 1000) | 1 | WB/ IFA | (61) |
| | (18/60) | 30 | (1/100) | 1 | WB | (24, 25) |
| | (18/132) | 13.6 | (3/203) | 1.5 | WB | (27) |
| | (13/55) | 23.6 | (4/36) | 11.1 | IFA | (51) |
| | (6/49) | 12.2 | | | IFA | (38) |
| Affective | (12/265) | 4.5 | (0/105) | 0 | IFA | (62) |
| | (12/285) | 4.2 | (0/200) | 0 | IFA | (36) |
| | (53/138) | 38 | (19/117) | 16 | WB | (37) |
| | (6/52) | 11.5 | (3/203) | 1.4 | WB | (27) |
| | (10/27) | 37 | | | IFA | (38) |
| Schizophrenia | (29/90) | 32 | (4/20) | 20 | WB | (43) |
| | (16/114) | 14 | (3/203) | 1.5 | WB | (27) |
| | (1/4) | 25 | | | IFA | (38) |
| CFS | (6/25) | 24 | | | WB | (26) |
| MS | (15/114) | 13 | (10/483) | 2.1 | IP/ IFA | (63) |
| HIV-positive | (36/460) | 7.8 | (11/540) | 2 | IFA | (60) |
| HIV-early | (61/751) | 8.1 | (10/483) | 2 | IP/ IFA | (61) |
| HIV-LAP | (34/244) | 14 | (10/483) | 2 | IP/ IFA | (63) |
| Schisto/ malaria | (19/193) | 9.8 | (10/483) | 2 | IP/ IFA | (63) |

Abbreviations: IFA, immunofluorescence assay; WB, Western immunoblot; IP, immunoprecipitation; CFS, chronic fatigue syndrome; MS, multiple sclerosis; HIV, human immunodeficiency virus; LAP, lymphadenopathy; Schisto/malaria, schistosomiasis or malaria.

To establish a correlation between immunoreactivity to BDV and the duration and severity of psychiatric disease, Bode et al. performed IFA on multiple serum samples taken at different times from 71 patients with various diagnoses including minor or major depression, paranoid psychosis, schizophrenia, anxiety disorder, and personality disorder (38). Overall, the prevalence of immunoreactivity to BDV was greater than 20%, a marked increase from the 2% to 4% found in an earlier study that assayed specimens from each patient at one time. Thirty-seven percent of patients with major depression, 25% with paranoid psychosis, and only 6% or fewer with reactive depression and other neurotic conditions were seropositive by day 17 of illness. Because BDV gene products have been identified in PBMC of infected rats (28), PBMC from patients with neuropsychiatric diseases were examined for viral antigens by fluorescence activated cell sorting analysis (39). Of the 70 patients tested, more than 40% were antigen carriers, twice the number predicted by the previous serologic survey.

Some similarities between particular animal models of BDV infection and schizophrenia have been reported. The subtle signs of disease caused by infection in neonatal rats (e.g., learning deficiencies, hyperactivity with CNS abnormalities including cerebellar disorganization and loss of dentate gyrus granule cells [32,33,40,41]) are consistent with a longstanding hypothesis that schizophrenia reflects an early brain insult (e.g., infection) resulting in abnormal brain development (42) and prompted investigation of the role of BDV infection in the pathogenesis of schizophrenia (43). A Western immunoblot assay based on BDV N, P, and matrix protein (M) purified from infected human neuroblastoma cells was used to examine sera from 90 schizophrenic patients and 20 healthy controls. Antibodies to one BDV protein (N, P, or M) were detected in 29 (32%) patients and four (20%) controls. Antibodies to two or more BDV proteins were detected in 13 (14.4%) patients and zero controls. Antibodies to M protein were found in 12 (13.3%) patients and zero controls. Immunoreactivity to two or more BDV proteins or M protein was significantly associated with abnormal brain morphology in magnetic resonance image analysis (MRI) and the clinical diagnosis of deficit syndrome (a schizophrenia subgroup characterized by social withdrawal, neurologic dysfunction, and neuroanatomic abnormalities). Similar findings indicated an association between antibodies reactive with BDV proteins and MRI evidence of cerebral atrophy in schizophrenic patients (44).

### Molecular Epidemiology (Table 2)

Interest in the potential role of BDV as a human pathogen and in BDV-infected animals as models for human neuropsychiatric diseases guided efforts to identify the infectious agent. Because of low viral productivity and tight association of BDV with plasma membranes, classic methods for virus isolation have been unsuccessful; however, BDV nucleic acids have been independently cloned from horse isolates by using a purely molecular subtractive cloning approach (45,46). The viral genome was subsequently cloned from viral particles (4) and nuclear extracts of infected cells (47). With the advent of viral sequence information, new diagnostic reagents

Table 2. Borna disease virus nucleic acid in patients with various diseases

| Disease | Tissue | Prevalence | | | | Divergence* | Ref. |
|---|---|---|---|---|---|---|---|
| | | Disease | (%) | Controls | (%) | | |
| Psychiatric | PBMC | (4/6) | 66.7 | (0/10) | 0 | 0-3.6 | (22) |
| (various) | PBMC | (5/12) | 41.7 | (0/23) | 0 | 0-4 | (27) |
| | PBMC | (22/60) | 37 | (8/172) | 4.7 | | (24, 25) |
| | PBMC-coculture | (3/32) | 9.4 | (0/5) | 0 | 0.07-0.83 | (23, 53) |
| Affective | PBMC | (1/3) | 33.3 | (0/23) | 0 | | (27) |
| | PBMC | (1/6) | 16.7 | (0/36) | 0 | | (51) |
| | PBMC | (0/9) | 0 | | | | (56) |
| Schizophrenia | PBMC | (7/11) | 63.6 | (0/23) | 0 | | (27) |
| | PBMC | (5/49) | 10.2 | (0/36) | 0 | | (51) |
| | PBMC | | | | | 4.2-9.3 | (54) |
| | Brain | (0/3) | 0 | (0/3) | 0 | | (55) |
| | CSF | (0/48) | 0 | (0/9) | 0 | | (55) |
| | PBMC | (0/9) | 0 | (0/9) | 0 | | (55) |
| | PBMC | (0/26) | 0 | | | | (56) |
| CFS | PBMC | (3/25) | 12 | | | 6.0-14 | (26) |
| Hippocampal sclerosis | Brain | (4/5) | 80 | | | | (53) |

Abbreviations: PBMC, peripheral blood mononuclear cells; CSF, cerebrospinal fluid; CFS, chronic fatigue syndrome.
*Divergence of P-gene nucleotide sequence from common BDV isolates (strain V [4] and He/80 [9]).

for BDV infection have been introduced, including recombinant proteins for serology as well as oligonucleotide primers and probes for molecular epidemiology.

The earliest experiments in BDV molecular epidemiology examined whether conserved virus sequences could be identified across several host species. Reverse transcription polymerase chain reaction (RT-PCR), as well as extensive experimental passage through rabbit and rat brains and cell lines from various species, was used to amplify and clone coding and noncoding sequences from virus strains divergent by over 50 years' growth in nature. Because of inaccuracies in viral RNA-dependent RNA polymerases, most single-stranded RNA viruses have sequence divergence of $10^3$ to $10^4$ per site per round of replication (48-50). Sequence analysis of BDV isolates showed a much lower rate of divergence. For N and P sequences, maximum variability was 4.1% at the nucleotide level and 1.5% at the predicted amino acid level (8). Similar sequence conservation was later found for sequences from naturally infected donkeys, sheep, and cats (9, H. Ludwig, pers. comm.). The extent to which sequence conservation in BDV represents enhanced polymerase fidelity, or more likely, selective environmental pressures is unknown.

Extending molecular analysis to human materials is both complex and controversial. If BDV infects neuropsychiatric disease patients, viral nucleic acids are present at lower concentrations in human brain and PBMC than in previously studied naturally or experimentally infected hosts because investigators who report isolation of BDV nucleic acid from human PBMC must use the highly sensitive technique of nested RT-PCR. Groups in Germany and Japan have detected viral nucleic acid in PBMC of patients with neuropsychiatric diseases: Bode and colleagues found BDV nucleic acids in four (66.7%) of six patients (22); Sauder and colleagues found BDV sequences in 13 (50%) of 26 neuropsychiatric patients (seven patients with schizophrenia, one with affective disorder, and five with other psychiatric disorders) (27); Kishi and co-workers found BDV nucleic acids in PBMC of 22 (37%) out of 60 neuropsychiatric patients (24) and eight (4.7%) out of 172 blood donor controls (25); Igata-Yi et al. detected BDV nucleic acids in six (10.9%) of 55 neuropsychiatric patients (five patients with schizophrenia, one with depression) versus zero of 36 blood donor controls (51); and Nakaya et al. reported BDV nucleic acid in three (12%) of 25 chronic fatigue syndrome patients (26). Additionally, BDV nucleic acids and immunoreactivity have been detected in the hippocampus of four of five North American patients with postmortem diagnosis of hippocampal sclerosis (52).

Genomic analyses of BDV isolates from human patients have yielded differing estimates of nucleotide sequence conservation (Table 2). Extensive conservation of the N and P open reading frames, consistent with previous findings in field and tissue culture isolates (8), has been reported (22,27). Infectious BDV has been isolated after cocultivation of PBMC from neuropsychiatric patients with a human oligodendroglial cell line (23). Sequences of these isolates were highly conserved with previously identified sequences (53); other groups have described greater sequence divergence (26,54). However, differences in levels of sequence conservation may reflect variations in methods for RT-PCR amplification rather than true strain differences (27).

In two reports RT-PCR did not yield evidence of BDV nucleic acid in neuropsychiatric disease patients (Table 2): No BDV nucleic acid was found by RT-PCR analysis of brain tissue, cerebrospinal fluid, or PBMC from patients with schizophrenia (55); similarly, no BDV nucleic acid was found in RT-PCR studies of PBMC from 26 patients with schizophrenia and nine with affective disorders (56).

### Special Considerations for BDV Diagnostics

Although a wide variety of assays (IFA, Western immunoblot, radioimmunoprecipitation, and enzyme-linked immunosorbent assay) and antigen preparations (infected cells, infected cell extracts, and recombinant proteins produced in prokaryotic or baculovirus systems) have been used for BDV serology, generally accepted standards for diagnosis of human BDV infection have not been established. Because only limited data concerning interassay comparability for serology within individual laboratories (and none between different laboratories) have been established, discrepancies between investigators may reflect differences in clinical populations, assay sensitivity, or other factors.

Similarly, agreement concerning methods for molecular diagnosis of BDV infection is limited. Most investigators use RT-PCR; some use a method sensitive to 100 to 300 copies of RNA template (nested RT-PCR, 80 to 100 cycles), while others use less sensitive methods (no nesting, 30 cycles). Only investigators using the more sensitive method have reported BDV gene products in human materials (PBMC, brain).

RT-PCR, particularly nested RT-PCR, is prone to artifacts because of inadvertent introduction of template from laboratory isolates or cross-contamination of samples. Because putative human isolates detected by nested RT-PCR are similar in sequence to known animal and tissue culture isolates, it has been argued that they represent low-level contaminants. However, the finding of sequence conservation is consistent with previous analyses of well-characterized isolates disparate by host species and geography (8,9) and cannot be used to discount the validity of positive nested RT-PCR results.

### Future Directions

The broad potential host range of BDV suggests that humans are targets for infection. Rodents with persistent BDV infection and minimal overt signs of disease and domestic animals and livestock could serve as vectors. Serum antibodies reactive with BDV have been detected in asymptomatic farmworkers exposed to ostriches with a Borna disease-like syndrome (57). Immunoreactivity to BDV and neurologic disturbances have been reported in a farm worker exposed to seropositive asymptomatic horses and sheep (58). Finally, although no human cases of disease have been linked to feline infection, there is evidence of BDV infection in house cats in Europe (59) and Japan (18). However, even though BDV could infect humans and is likely to do so, a number of questions remain unanswered: The sources and routes of potential human infection are not clear, no detailed epidemiology has been done in animal populations, and transmission from domestic animals to humans has not been demonstrated.

Viral gene products are readily detected in CNS of natural and experimental hosts without such sensitive methods as nested RT-PCR. The need to use sensitive methods to detect BDV nucleic acid in humans indicates that the virus is present only at low levels. Thus, if BDV can be implicated as a factor in human neuropsychiatric disease, mechanisms for pathogenesis may be different from those found in other natural and experimental hosts. Although a higher prevalence of markers for BDV infection has been reported in neuropsychiatric patients than in controls, no single neuropsychiatric disease has been correlated with BDV infection. Efforts to link BDV with neuropsychiatric disease have not used accepted epidemiology standards.

To rigorously address the issues of BDV epidemiology and pathogenesis, multicenter groups in Europe and the United States are collaborating to collect and perform blinded analysis of human clinical materials by standardized methods and reagents. The objectives of these projects will be to determine the prevalence of serum antibodies to BDV in patients and controls and the extent to which various assays for antibodies are in accord, the prevalence of BDV nucleic acids in brains and PBMC of patients and controls, and the correlation between antibodies to BDV or viral nucleic acids and a particular neuropsychiatric disease.

### Acknowledgments

The Laboratory for Neurovirology is supported by a grant from the National Institutes of Health (NS29425), as well as awards from the Wayne and Gladys Valley Foundation, Cure Autism Now Foundation, and Lucille P. Markey Trust.

Dr. Hatalski is a postdoctoral fellow in the Department of Anatomy & Neurobiology, University of California, Irvine, California, USA.

### References

1. de la Torre JC. Molecular biology of Borna disease virus: prototype of a new group of animal viruses. J Virol 1994;68:7669-75.
2. Schneemann A, Schneider PA, Lamb RA, Lipkin WI. The remarkable coding strategy of Borna disease virus: a new member of the nonsegmented negative strand RNA viruses. Virology 1995;210:1-8.
3. Briese T, de la Torre JC, Lewis A, Ludwig H, Lipkin WI. Borna disease virus, a negative-strand RNA virus, transcribes in the nucleus of infected cells. Proc Natl Acad Sci USA 1992;89:11486-9.
4. Briese T, Schneemann A, Lewis AJ, Park YS, Kim S, Ludwig H, Lipkin WI. Genomic organization of Borna disease virus. Proc Natl Acad Sci USA 1994;91:4362-6.
5. Schneemann A, Schneider PA, Kim S, Lipkin WI. Identification of signal sequences that control transcription of Borna disease virus, a nonsegmented negative-strand RNA virus. J Virol 1994;68:6514-22.
6. Cubitt B, Oldstone C, Valcarcel V, de la Torre JC. RNA splicing contributes to the generation of mature mRNAs of Borna disease virus, a non-segmented negative strand RNA virus. Virus Res 1994;34:69-79.
7. Schneider PA, Schneemann A, Lipkin WI. RNA splicing in Borna disease virus, a nonsegmented, negative-strand RNA virus. J Virol 1994;68:5007-12.

8. Schneider PA, Briese T, Zimmermann W, Ludwig H, Lipkin WI. Sequence conservation in field and experimental isolates of Borna disease virus. J Virol 1994;68:63-8.
9. Binz T, Lebelt J, Niemann H, Hagenau K. Sequence analyses of the p24 gene of Borna disease virus in naturally infected horse, donkey and sheep. Virus Res 1994;34:281-9.
10. Ludwig H, Bode L, Gosztonyi G. Borna disease: a persistent disease of the central nervous system. Prog Med Virol 1988;35:107-51.
11. Richt JA, VandeWoude S, Zink MC, Clements JE, Herzog S, Stitz L, et al. Infection with Borna disease virus: molecular and immunobiological characterization of the agent. Clin Inf Dis 1992;14:1240-50.
12. Solbrig MV, Fallon JH, Lipkin WI. Behavioral disturbances and pharmacology of Borna disease virus. Curr Top Microbiol Immunol 1995;190:93-102.
13. Narayan O, Herzog S, Frese K, Scheefers H, Rott R. Behavorial disease in rats caused by immunopathological responses to persistent Borna virus in the brain. Science 1983;220:1401-3.
14. Stitz L, Dietzschold B, Carbone KM. Immunopathogenesis of Borna disease. Curr Top Microbiol Immunol 1995;190:75-92.
15. Rott R, Becht H. Natural and experimental Borna disease in animals. Curr Top Microbiol Immunol 1995;190:17-30.
16. Kao M, Hamir AN, Rupprecht CE, Fu AF, Shankar V, Koprowski H, Dietzschold B. Detection of antibodies against Borna disease virus in sera and cerebrospinal fluid of horses in the USA. Vet Rec 1993;132:241-4.
17. Nakamura Y, Kishi M, Nakaya T, Asahi S, Tanaka H, Sentsui H, et al. Demonstration of Borna disease virus RNA in peripheral blood mononuclear cells from healthy horses in Japan. Vaccine 1995;13:1076-9.
18. Nakamura Y, Asahi S, Nakaya T, Bahmani MK, Saitoh S, Yasui K, et al. Demonstration of borna disease virus RNA in peripheral blood mononuclear cells derived from domestic cats in Japan. J Clin Microbiol 1996;34:188-91.
19. Gosztonyi G, Ludwig H. Borna disease-neuropathology and pathogenesis. Curr Top Microbiol Immunol 1995;190:39-73.
20. Morales JA, Herzog S, Kompter C, Frese K, Rott R. Axonal transport of Borna disease virus along olfactory pathways in spontaneously and experimentally infected rats. Med Microbiol Immunol 1988;177:51-68.
21. Bode L. Human infections with Borna disease virus and potential pathologic implications. Curr Top Microbiol Immunol 1995;190:103-30.
22. Bode L, Zimmermann W, Ferszt R, Steinbach F, Ludwig H. Borna disease virus genome transcribed and expressed in psychiatric patients. Nature Med 1995;1:232-6.
23. Bode L, Dürrwald R, Rantam FA, Ferszt R, Ludwig H. First isolates of infectious human Borna disease virus from patients with mood disorders. Mol Psychiatry 1996;1:200-12.
24. Kishi M, Nakaya T, Nakamura Y, Zhong Q, Ikeda K, Senjo M, et al. Demonstration of human Borna disease virus RNA in human peripheral blood mononuclear cells. FEBS Letters 1995;3645:293-7.
25. Kishi M, Nakaya T, Nakamura Y, Kakinuma M, Takahashi TA, Sekiguchi S, et al. Prevalence of Borna disease virus RNA in peripheral blood mononuclear cells from blood donors. Med Microbiol Immunol 1995;184:135-8.
26. Nakaya T, Takahashi H, Nakamura Y, Asahi S, Tobiume M, Kuratsune H, et al. Demonstration of Borna disease virus RNA in peripheral blood mononuclear cells derived from Japanese patients with chronic fatigue syndrome. FEBS Letters 1996;378:145-9.
27. Sauder C, Muller A, Cubitt B, Maer J, Steinmetz J, Trabert W, et al. Detection of Borna disease virus (BDV) antibodies and BDV RNA in psychiatric patients: evidence for high sequence conservation of human blood-derived BDV RNA. J Virol 1996;70:7713-24.
28. Sierra-Honigmann AM, Rubin SA, Estafanous MG, Yolken RH, Carbone KM. Borna disease virus in peripheral blood mononuclear and bone marrow cells of neonatally and chronically infected rats. J Neuroimmunol 1993;45:31-6.
29. Abildgaard PC. Pferde-und Vieharzt in einem kleinen Auszuge; oder, Handbuch von den gewöhnlichsten Krankheiten der Pferde, des Hornviehes, der Schafe und Schweine, sammt der bequemsten und wohl-feilesten Art sie zu heilen. Zum Gebrauch des Land-manns. Wien: Johann Thomas Edlen von Trattnern, 1785.
30. Carbone K, Duchala C, Griffin J, Kincaid A, Narayan O. Pathogenesis of Borna disease in rats: evidence that intra-axonal spread is the major route for virus dissemination and the determination for disease incubation. J Virol 1987;61:3431-40.
31. Solbrig MV, Koob GF, Joyce JN, Lipkin WI. A neural substrate of hyperactivity in Borna disease: changes in dopamine receptors. Virology 1996;222:332-8.
32. Dittrich W, Bode L, Ludwig H, Kao M, Schneider K. Learning deficiencies in Borna disease virus-infected but clinically healthy rats. Biol Psychiatry 1989;26:818-28.
33. Carbone K, Park S, Rubin S, Waltrip R, Vogelsang G. Borna disease: association with a maturation defect in the cellular immune response. J Virol 1991;65:6154-64.
34. Sprankel H, Richarz K, Ludwig H, Rott R. Behavior alterations in tree shrews induced by Borna disease virus. Med Microbiol Immunol 1978;165:1-18.
35. Stitz L, Krey H, Ludwig H. Borna disease in rhesus monkeys as a model for uveo-cerebral symptoms. J Med Virol 1980;6:333-40.
36. Rott R, Herzog S, Fleischer B, Winokur A, Amsterdam J, Dyson W, Koprowski H. Detection of serum antibodies to Borna disease virus in patients with psychiatric disorders. Science 1985;228:755-6.
37. Fu ZF, Amsterdam JD, Kao M, Shankar V, Koprowski H, Dietzschold B. Detection of Borna disease virus-reactive antibodies from patients with affective disorders by western immunoblot technique. J Affect Disord 1993;27:61-8.
38. Bode L, Ferszt R, Czech G. Borna disease virus infection and affective disorders in man. Arch Virol 1993;[Suppl] 7:159-67.
39. Bode L, Steinbach F, Ludwig H. A novel marker for Borna disease virus infection. Lancet 1994;343:297-8.
40. Bautista JR, Schwartz GJ, de la Torre JC, Moran TH, Carbone KM. Early and persistent abnormalities in rats with neonatally acquired Borna disease virus infection. Brain Res Bull 1994;34:31-40.

41. Bautista JR, Rubin SA, Moran TH, Schwartz GJ, Carbone KM. Developmental injury to the cerebellum following perinatal Borna disease virus infection. Brain Res Dev Brain Res 1995;90:45-53.
42. Yolken RH, Torrey EF. Viruses, schizophrenia, and bipolar disorder. Clin Microbiol Rev 1995;8:131-45.
43. Waltrip II RW, Buchanan RW, Summerfelt A, Breier A, Carpenter WT, Bryant NL, et al. Borna disease virus and schizophrenia. Psychiatry Res 1995;56:33-44.
44. Bechter K, Bauer M, Estler HC, Herzog S, Schuttler R, Rott R. Expanded nuclear magnetic resonance studies in Borna disease virus seropositive patients and control probands. Nervenarzt 1994;65:169-74.
45. Lipkin WI, Travis G, Carbone K, Wilson M. Isolation and characterization of Borna disease agent cDNA clones. Proc Natl Acad Sci USA 1990;87:4184-8.
46. VandeWoude S, Richt J, Zink M, Rott R, Narayan O, Clements J. A Borna virus cDNA encoding a protein recognized by antibodies in humans with behavioral diseases. Science 1990;250:1276-81.
47. Cubitt B, Oldstone C, de la Torre JC. Sequence and genome organization of Borna disease virus. J Virol 1994;68:1382-96.
48. Holland J, Spindler K, Horodyski F, Grabau E, Nichol S, VandePol S. Rapid evolution of RNA genomes. Science 1982;215:1577-85.
49. Holland JJ, de la Torre JC, Steinhauer DA. RNA virus populations as quasispecies. Curr Top Microbiol Immunol 1992;176:1-20.
50. Morse SS. The evolutionary biology of viruses. New York: Raven, 1994.
51. Igata-Yi R, Kazunari Y, Yoshiki K, Takemoto S, Yamasaki H, Matsuoka M, Miyakawa T. Borna disease virus and consumption of raw horse meat. Nat Med 1996;2:948-9.
52. de la Torre JC, Gonzalez-Dunia D, Cubitt B, Mallory M, Mueller-Lantzsch N, Grasser F, et al. Detection of Borna disease virus antigen and RNA in human autopsy brain samples from neuropsychiatric patients. Virology 1996;223:272-82.
53. de la Torre JC, Bode L, Durrwald R, Cubitt B, Ludwig H. Sequence characterization of human Borna disease virus. Virus Res 1996;44:33-44.
54. Kishi M, Arimura Y, Ikuta K, Shoya Y, Lai PK, Kakinuma M. Sequence variability of Borna disease virus open reading frame II found in human peripheral blood mononuclear cells. J Virol 1996;70:635-40.
55. Sierra-Honigman AM, Carbone KM, Yolken RH. Polymerase chain reaction (PCR) search for viral nucleic acid sequences in schizophrenia. Br J Psychiatry 1995;166:55-60.
56. Richt JA, Alexander RC, Herzog S, Hooper DC, Kean R, Spitzen S, et al. Failure to associate human psychiatric disorders with Borna disease virus infection. In press.
57. Weisman Y, Huminer D, Malkinson M, Meir L, Kliche S, Lipkin WI, Pitlik S. Borna disease virus antibodies among workers exposed to infected ostriches. Lancet 1994;344:1232-3.
58. Bechter K, Schüttler R, Herzog S. Case of neurological and behavioral abnormalities: due to Borna disease virus encephalitis? Psychiatry Res 1992;42:193-6.
59. Lundgren A-L, Czech G, Bode L, Ludwig H. Natural Borna disease in domestic animals other than horses and sheep. J Vet Med 1993;40:298-303.
60. Bode L, Riegel S, Ludwig H, Amsterdam J, Lange W, Koprowski H. Borna disease virus-specific antibodies in patients with HIV Infection and with mental disorders. Lancet 1988;ii:689.
61. Rott R, Herzog S, Bechter K, Frese K. Borna disease, a possible hazard for man. Arch Virol 1991;118:143-9.
62. Amsterdam J, Winokur A, Dyson W, Herzog S, Gonzalez F, Rott R, Koprowski H. Borna Disease Virus: a possible etiologic factor in human affective disorders. Arch Gen Psychiatry 1985;42:1093-6.
63. Bode L, Riegel S, Lange W, Ludwig H. Human infections with Borna disease virus: seroprevalence in patients with chronic diseases and healthy individuals. J Med Virol 1992;36:309-15.
